# Thermoelectric and Transport Properties of Permingeatite Cu_3_SbSe_4_ Prepared Using Mechanical Alloying and Hot Pressing

**DOI:** 10.3390/ma14051116

**Published:** 2021-02-27

**Authors:** Go-Eun Lee, Il-Ho Kim

**Affiliations:** Department of Materials Science and Engineering, College of Engineering, Korea National University of Transportation, Chungju 27469, Korea; leege0205@ut.ac.kr

**Keywords:** thermoelectric, permingeatite, mechanical alloying, hot pressing

## Abstract

Permingeatite (Cu_3_SbSe_4_) is a promising thermoelectric material because it has a narrow band gap, large carrier effective mass, and abundant and nontoxic components. Mechanical alloying (MA), which is a high-energy ball mill process, has various advantages, e.g., segregation/evaporation is not required and homogeneous powders can be prepared in a short time. In this study, the effects of MA and hot-pressing (HP) conditions on the synthesis of the Cu_3_SbSe_4_ phase and its thermoelectric properties were evaluated. The electrical conductivity decreased with increasing HP temperature, while the Seebeck coefficient increased. The power factor (PF) was 0.38–0.50 mW m^−1^ K^−2^ and the thermal conductivity was 0.76–0.78 W m^−1^ K^−1^ at 623 K. The dimensionless figure of merit, *ZT*, increased with increasing temperature, and a reliable and maximum *ZT* value of 0.39 was obtained at 623 K for Cu_3_SbSe_4_ prepared using MA at 350 rpm for 12 h and HP at 573 K for 2 h.

## 1. Introduction

Thermoelectric conversion techniques have been studied for applications in solid-state cooling and power generation because they can convert thermal energy directly to electrical energy and vice versa. In particular, thermoelectric power generation technology has received attention because it is the only way to directly convert thermal energy to electrical energy; in addition, thermal energy sources, such as solar heat and industrial and automotive waste heat, are abundant. The energy conversion efficiency of a thermoelectric material is evaluated using its dimensionless figure of merit, defined as *ZT = α*^2^*σκ*^−1^*T*, where *α* is the Seebeck coefficient, *σ* is the electrical conductivity, *κ* is the thermal conductivity, and *T* is the absolute temperature. According to the above equation, excellent thermoelectric materials require a large power factor (PF = *α^2^σ*) and low thermal conductivity at a suitable application temperature. Many studies have been conducted to improve *ZT* values, for example, by increasing the electrical transport properties by optimizing the carrier concentration or by reducing the thermal conductivity through the vibration of fillers in the voids [1,2]. Most thermoelectric materials, such as Bi_2_Te_3_, PbTe, and skutterudite compounds, which exhibit good performance, contain toxic heavy metals or rare elements. Recently, the development of thermoelectric materials composed of nontoxic and low-cost elements has been considered [3]. Cu-based chalcogenides are attracting attention as promising thermoelectric materials.

Cu_3_SbSe_4_ (permingeatite) exhibits a zinc-blende-type tetragonal structure with the space group I$\bar{4}2$ m. This compound is suitable as a *p*-type thermoelectric material at intermediate temperatures [4] because of its small band-gap energy (0.29–0.4 eV) and large carrier effective mass (≈1.1 m_e_) [5,6]. In most studies, Cu_3_SbSe_4_ compounds have been synthesized using a direct melting reaction of elements [7,8,9], but this method requires a low heating rate and long annealing time for homogenization. Mechanical alloying (MA) does not require the volatilization of chalcogen elements and maintains the homogeneity of the constituent elements [10,11]. In addition, it is suitable for large-scale production as homogeneous samples can be obtained in a short time [12]. In our previous study [13], Cu_3_SbS_4_ (famatinite) with the same crystal structure was successfully prepared using the MA–hot-pressing (HP) process as a solid-state method. In this study, Cu_3_SbSe_4_ was synthesized using MA and consolidated using HP. The phase transition and thermoelectric performance according to the MA and HP conditions were examined.

## 2. Experimental Procedure

For the synthesis, Cu (purity 99.9%, <45 μm powder, Kojundo, Japan), Sb (purity 99.999%, <75 μm powder, Kojundo, Japan), and Se (purity 99.9%, <10 μm powder, Kojundo, Japan) were weighed in a stoichiometric ratio and loaded into a hardened steel jar with steel balls of 5 mm in diameter at a ball-to-powder weight ratio of 20. MA was performed using planetary ball milling (Pulverisette5, Fritsch, Germany) at 350 rpm for 6–24 h in an Ar atmosphere. The synthesized powders were hot-pressed in a cylindrical graphite die at 523–623 K for 2 h under 70 MPa in a vacuum.

The phases of the MA powders and the HP compacts were analyzed using X-ray diffraction (XRD; D2-Phaser, Bruker, Germany) with Cu K_α_ radiation (*λ* = 0.15405 nm), and Rietveld refinement (TOPAS, Bruker, Germany) was performed to estimate the lattice constants. The weight changes and phase transitions were analyzed using thermogravimetry and differential scanning calorimetry (TG–DSC; TG/DSC1, Mettler Toledo, Columbus, OH, USA) in an Ar atmosphere. Field-emission scanning electron microscopy (FESEM; JSM-7610F, Jeol, Japan) was used in conjunction with energy-dispersive X-ray spectroscopy (EDS; X-Max50, Oxford Instruments, Oxford, UK) to observe the microstructure and analyze the compositions and elemental distributions.

The hot-pressed compact was cut into a disc shape with dimensions of 10 mm (diameter) × 1 mm (thickness) for the thermal conductivity and Hall measurements and into a rectangular shape with dimensions of 3 × 3 × 9 mm^3^ for both the Seebeck coefficient and electrical conductivity measurements. The charge transport parameters (Hall coefficient, carrier concentration, and mobility) were measured using a Hall 7065 (Keithley, Cleveland, OH, USA) system. The Seebeck coefficient and electrical conductivity were measured using ZEM-3 (Ulvac-Riko, Kanagawa, Japan) equipment in a He atmosphere. The thermal conductivity was estimated from the specific heat, density, and thermal diffusivity measured using a laser flash TC-9000H (Ulvac-Riko) system in a vacuum. The PF and *ZT* values were evaluated at temperatures ranging from 323 K to 623 K.

## 3. Results and Discussion

**Figure 1:**
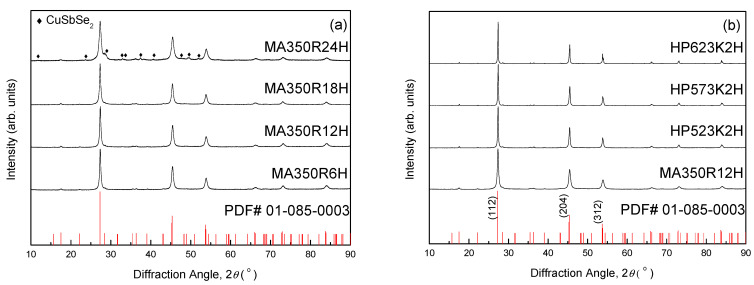
X-ray diffraction (XRD) patterns for (**a**) synthetic powders of Cu_3_SbSe_4_ for different mechanical alloying (MA) times and (**b**) sintered specimens with different hot-pressing (HP) temperatures.

Figure 1 shows the XRD patterns of the synthetic Cu_3_SbSe_4_ prepared at different MA–HP conditions. Cu_3_SbSe_4_ (permingeatite) was produced after MA for 6 h (MA350R6H), and no secondary phases were identified after MA for 18 h (MA350R18H). However, the secondary phase of CuSbSe_2_ (příbramite) was formed after MA for 24 h (MA350R24H). Patil et al. [14] suggested that MA can change the relative thermodynamic stabilities of the different phases owing to the introduction of mechanical energy. Zhou et al. [15] reported that the mechanical collision energy, which is the main driving force of chemical reactions in ball-milling systems, can destroy structural periodicities and strengthen/join broken chemical bonds, free ions of electrons, and create new surfaces. In addition, when the mechanical energy is high enough, chemical reactions can be induced to reduce the free energy of the materials. Therefore, it was considered that excess collision energy due to a long milling time resulted in the decomposition of the synthesized Cu_3_SbSe_4_ phase, and these results have also been reported for other thermoelectric materials, such as higher manganese silicides [16,17].

**Figure 2:**
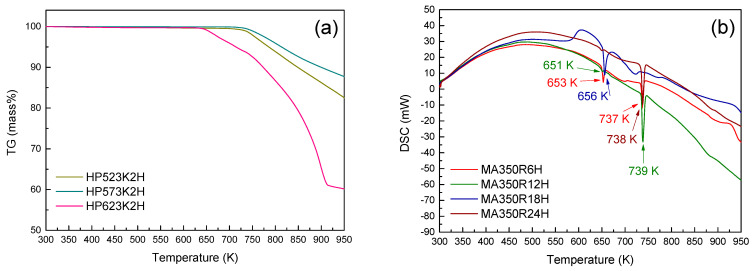
Thermogravimetry (TG) (**a**) and differential scanning calorimetry (DSC) (**b**) analyses for the mechanically alloyed Cu_3_SbSe_4_ powders.

TG–DSC analyses were performed to confirm the phase transformation, as shown in Figure 2. The endothermic peaks near 650 K of the MA powders were possibly related to the peritectic reaction (Cu_2_Se + Cu_0.43_Sb_0.14_Se_0.43_ → Cu_3_SbSe_4_) [18,19]. The endothermic peaks near 735 K were attributed to the melting of Cu_3_SbSe_4_ [20,21]. In this study, the optimal MA condition was determined to be 350 rpm for 12 h.

Figure 1b presents the XRD analysis results of the samples that were sintered at different HP temperatures. In the sample designation, the numbers refer to the HP temperature and time; for example, HP523K2H indicates a specimen in which MA350R12H powder was hot-pressed at 523 K for 2 h. The diffraction peaks of all the HP specimens were well matched with the standard diffraction data for Cu_3_SbSe_4_ (PDF #01-085-0003), which implied that the permingeatite phase was maintained after HP and no secondary phases were identified. As shown in Table 1, regardless of the HP temperature, all specimens had similar lattice constants (*a* = 0.5646–0.5650 nm and *c* = 1.124 nm), corresponding to the reported data [6,22]. The FWHM of the (112) plane decreased from 0.355° for MA350R12H to 0.171–0.234° for the HP specimens. The FWHM stands for the full width at half maximum of the diffraction peak and provides a lot of crystallographic information. The broad diffraction peaks of the MA powder were attributed to the fine crystallite size, and the diffraction peaks after the HP became narrower due to grain growth and enhanced crystallinity.

**Figure 3:**
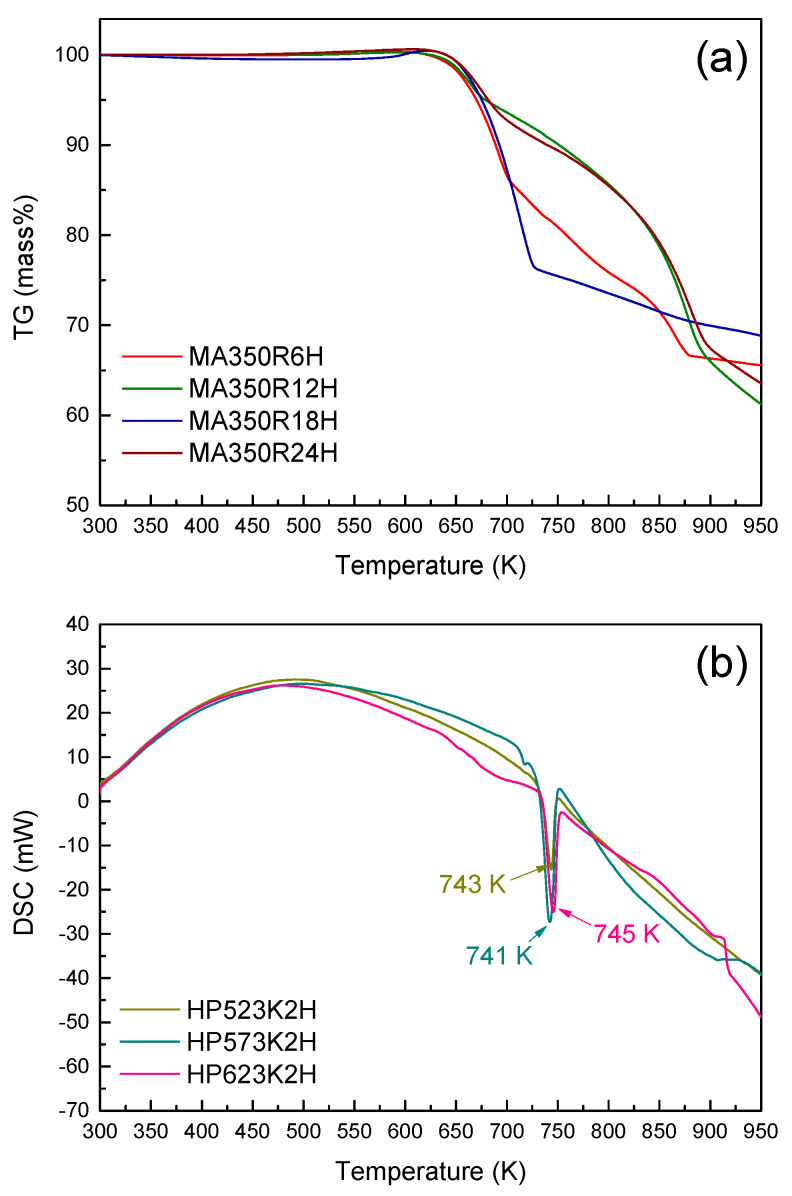
TG (**a**) and DSC (**b**) curves for the hot-pressed Cu_3_SbSe_4_ specimens.

The TG–DSC curves of the HP specimens are presented in Figure 3. The endothermic peaks at 735 K corresponded to the melting point of Cu_3_SbSe_4_, and the Cu_3_SbSe_4_ phase was stable during the HP because no phase transformation was observed up to this temperature.

**Figure 4:**
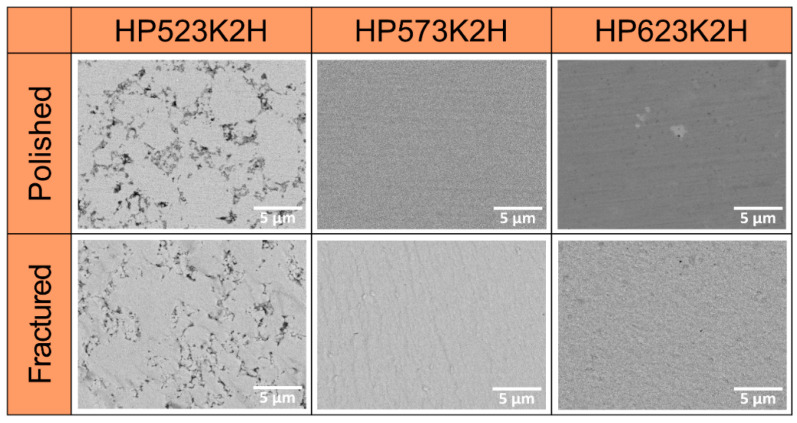
Field-emission scanning electron microscopy (FESEM) images of Cu_3_SbSe_4_ with different HP temperatures.

**Figure 5:**
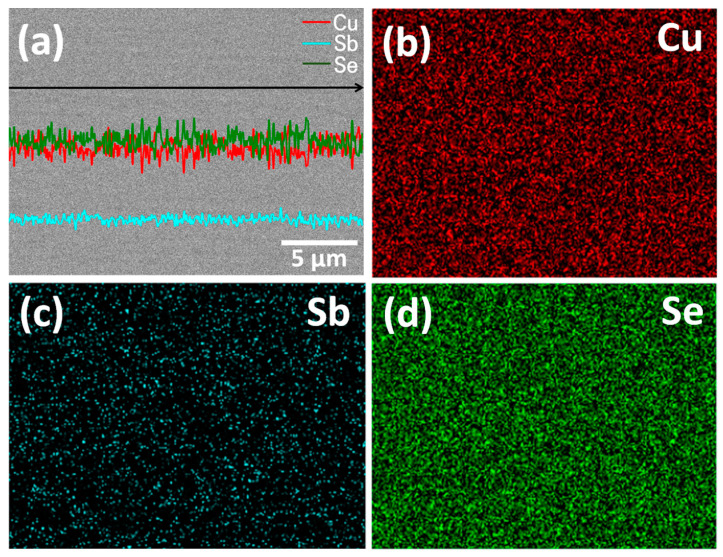
Energy-dispersive X-ray spectroscopy (EDS) analyses of the hot-pressed Cu_3_SbSe_4_ (HP573K2H): (**a**) line scans and elemental maps of (**b**) Cu, (**c**) Sb and (**d**) Se.

Figure 4 shows the FESEM images of Cu_3_SbSe_4_ prepared at different HP temperatures. The microstructure of HP523K2H was porous and less dense, which was consistent with its low relative density, as listed in Table 1. On the other hand, HP573K2H and HP623K2H had few cracks and pores, and their densities were higher than 97% of the theoretical density (5.86 g cm^−3^) [22,23,24]. The EDS images for HP573K2H are presented in Figure 5. Cu, Sb, and Se were homogeneously distributed without the formation of secondary phases and segregation.

**Figure 6:**
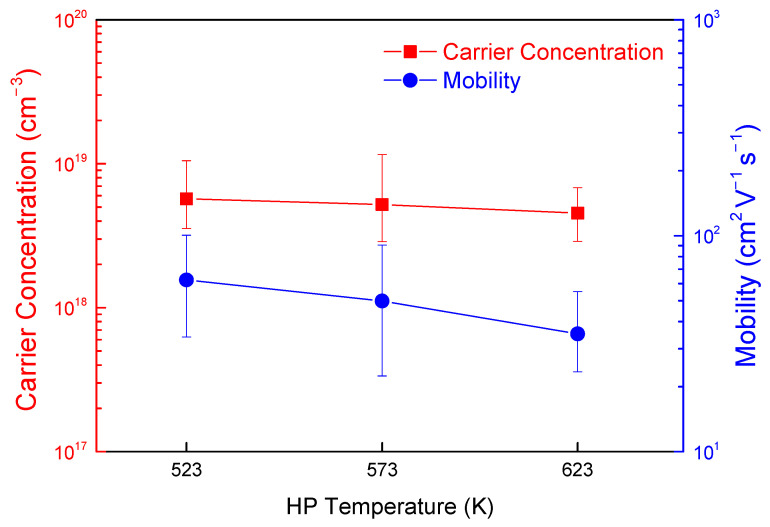
Variation of carrier concentration and mobility for Cu_3_SbSe_4_ with different HP temperatures.

Figure 6 presents the variation in the charge transport properties of Cu_3_SbSe_4_ prepared at different HP temperatures. Hall coefficient measurements revealed that all the Cu_3_SbSe_4_ specimens were *p*-type semiconductors with a carrier (hole) concentration of (4.5–5.7) × 10^18^ cm^−3^. In the Cu_3_SbSe_4_ system, Cu vacancies are easily formed due to the low formation energy and contribute to *p*-type behavior [25], which is often observed in other Cu chalcogenides [5]. Undoped Cu_3_SbSe_4_ has been reported as an intrinsic semiconductor with a carrier concentration of ~10^18^ cm^−3^ [5,6,7,26]. The carrier mobility values were 35–62 cm^2^ V^−1^ s^−1^, which decreased slightly with increasing HP temperature.

**Figure 7:**
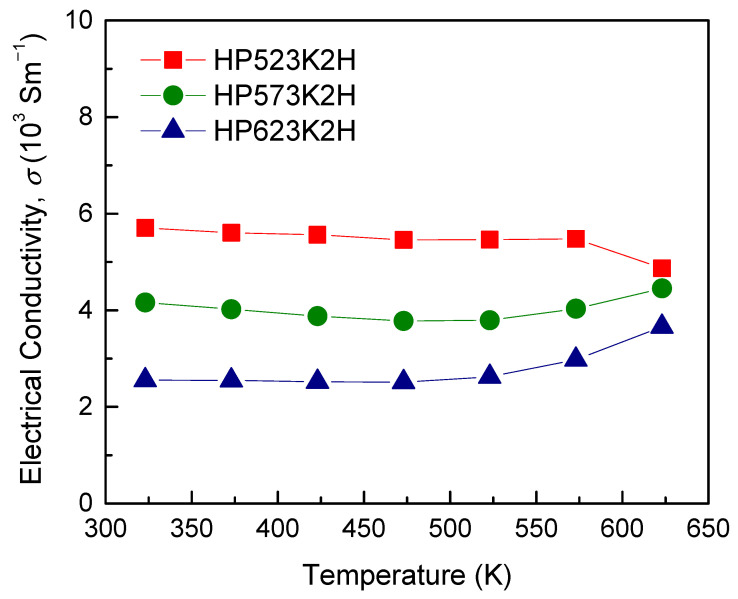
Temperature dependence of the electrical conductivity for Cu_3_SbSe_4_.

Figure 7 shows the electrical conductivity of Cu_3_SbSe_4_ prepared at different HP temperatures. In general, the electrical conductivity increases with increasing temperature for a non-degenerate semiconductor. Li et al. [22] reported that the electrical conductivity decreases with increasing temperature and then increases, reaching a minimum at a certain temperature, suggesting that Cu_3_SbSe_4_ is partially degenerate. The electrical conductivity decreased with increasing HP temperature, which was attributed to the decreased carrier concentration and mobility, as shown in Figure 6. The HP temperature can lead to fewer interfaces and pores, which results from the grain growth and densification, but changes the electronic transport properties, such as the carrier concentration and mobility. In this study, as the HP temperature increased, the carrier concentration could change because of the volatilization of Se with a high vapor pressure, and Se vacancies are likely to act as *n*-type dopants, thus decreasing the carrier concentration [20,25]. The electrical conductivities for all the specimens were (2.55–5.71) × 10^3^ S m^−1^ at 323 K and (3.66–4.87) × 10^3^ S m^−1^ at 623 K, indicating a low temperature dependence. Wei et al. [6] obtained 4.7 × 10^3^ S m^−1^ at 323 K and 9.5 × 10^3^ S m^−1^ at 673 K, exhibiting non-degenerate semiconducting behavior, and Zhao et al. [7] reported 2.3 × 10^3^ S m^−1^ at 323 K and 6.3 × 10^3^ S m^−1^ at 650 K.

**Figure 8:**
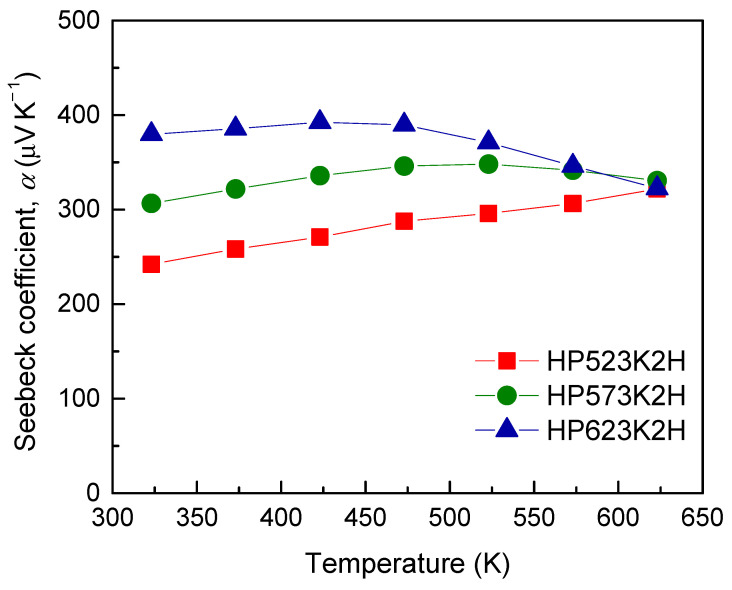
Temperature dependence of the Seebeck coefficient for Cu_3_SbSe_4_.

Figure 8 presents the Seebeck coefficient of Cu_3_SbSe_4_ prepared at different HP temperatures. The signs of the Seebeck coefficient were in good agreement with those of the Hall coefficient, which confirmed that the major charge carriers were holes (*p*-type conduction). Except for that of the HP523K2H specimen, the Seebeck coefficients of other specimens increased with increasing temperature and thereafter decreased due to an intrinsic transition, showing a maximum at a certain temperature (intrinsic transition temperature), which shifted to higher temperatures when the material had a broader bandgap energy and/or higher carrier concentration. In this study, as the HP temperature increased, the intrinsic transition temperature decreased. The Seebeck coefficient is expressed as *α* *=* (8*π*^2^*k_B_*^2^*T*/3*eh*^2^)m^*^(*π/*3*n*)^2/3^ (*k*_B_: Boltzmann constant, *e*: electron charge, *h*: Planck’s constant, m^*^: effective carrier mass, and *n*: carrier concentration), which indicates that the Seebeck coefficient is inversely proportional to the carrier concentration. Therefore, the Seebeck coefficient could increase with increasing HP temperature owing to a reduction in the carrier concentration, as shown by the variation in the electrical conductivity. Zhao et al. [7] obtained Seebeck coefficients of 405 μV K^−1^ at 300 K and 291 μV K^−1^ at 650 K for Cu_3_SbSe_4_ prepared via a melting process and spark plasma sintering (SPS). Kumar et al. [6] reported the Seebeck coefficient of undoped Cu_3_SbSe_4_ to be 347 μV K^−1^ at room temperature, which decreased with increasing temperature. In this study, the Seebeck coefficients of all specimens were 242–380 μV K^−1^ at 323 K and 321–330 μV K^−1^ at 623 K.

**Figure 9:**
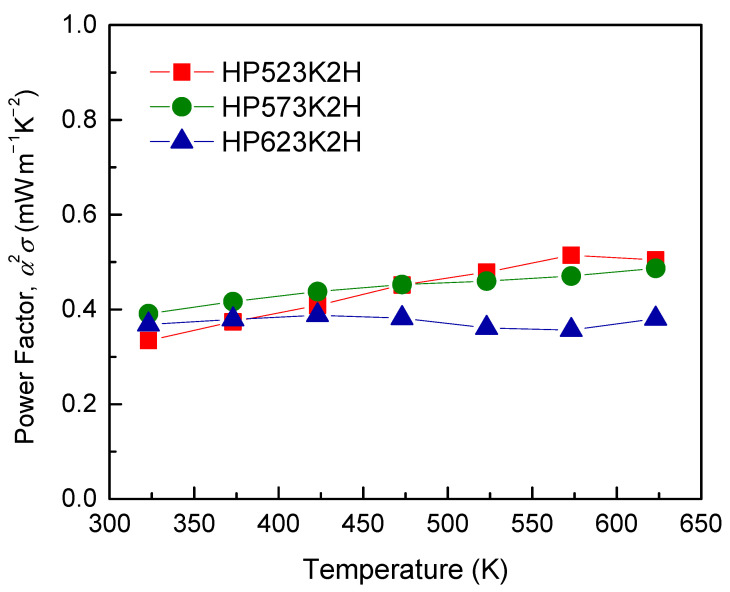
Temperature dependence of the power factor for Cu_3_SbSe_4_.

The PF of Cu_3_SbSe_4_ is shown in Figure 9. The maximum PF value of approximately 0.5 mW m^−1^ K^−2^ was obtained in the temperature range of 573–623 K for the HP523K2H and HP573K2H specimens. However, HP623K2H exhibited low electrical conductivity and a low Seebeck coefficient, resulting in the lowest PF value at high temperatures.

**Figure 10:**
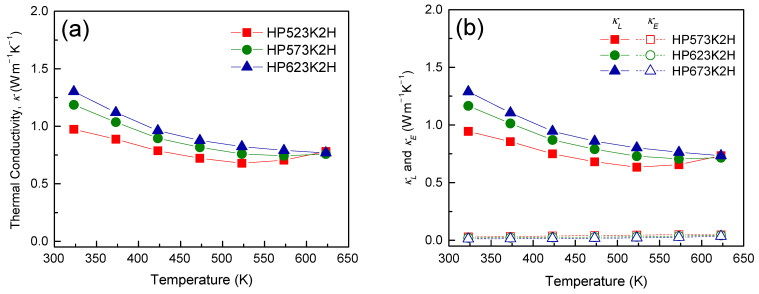
Temperature dependence of the thermal conductivity for Cu_3_SbSe_4_: (**a**) total thermal conductivity and (**b**) lattice and electronic thermal conductivities.

Figure 10 shows the thermal conductivity of Cu_3_SbSe_4_ prepared at different HP temperatures. To determine the thermal conductivity, a specific heat (*c_p_*) of 0.32 J g^−1^ K^−1^ was used [27]. Li et al. [23] obtained the values of *c_p_* via DSC analysis in the temperature range from 300 to 500 K, and the average value of *c_p_* was set to 0.33 J g^−1^ K^−1^. Zhou et al. [28] calculated the *c_p_* to be 0.318 J g^−1^ K^−1^ using the Dulong–Petit law. The thermal conductivity is a combination of the contributions from the lattice vibrations (*κ_L_*: lattice thermal conductivity) and the charge carrier transport (*κ_E_*: electronic thermal conductivity), which can be separated using the Wiedemann–Franz law (*κ_E_* = *LσT*, *L*: Lorenz number). The Lorenz number can be obtained using the equation *L* (10^−8^ V^2^ K^−2^) = 1.5 + exp(−|α|/116) [29], and in this study, it ranged from (1.54–1.62) × 10^−8^ V^2^ K^−2^ at 323 K to 1.56 × 10^−8^ V^2^ K^−2^ at 623 K. The estimated Lorenz numbers at 323 K for each specimen are summarized in Table 1. The thermal conductivity of Cu_3_SbSe_4_ was 0.97–1.30 W m^−1^ K^−1^ at 323 K and 0.76–0.78 W m^−1^ K^−1^ at 623 K, and there was little contribution from *κ_E_*. These *κ* values were much lower than 2.27–3.19 W m^−1^ K^−1^ at 300 K for the Cu_3_SbSe_4_ prepared using the melting and sintering process [5,20,30]. In addition, the *κ* values were similar to or lower than 1.1 W m^−1^ K^−1^ at 673 K for Cu_2.95_SbSe_4_ prepared using MA–SPS [6] and 0.91–1.15 W m^−1^ K^−1^ at 300–570 K for Cu_3_SbSe_4_ nanoparticles prepared using the rapid-injection route and HP [31]. This might be attributable to the reduced *κ_L_* due to enhanced phonon scattering through the introduction of a large number of grain boundaries during MA. The theoretical minimum *κ_L_* for Cu_3_SbSe_4_ was reported to be 0.47 W m^−1^ K^−1^ [6,7], and thus, *κ_L_* can be further reduced through the formation of solid solutions or doping.

**Figure 11:**
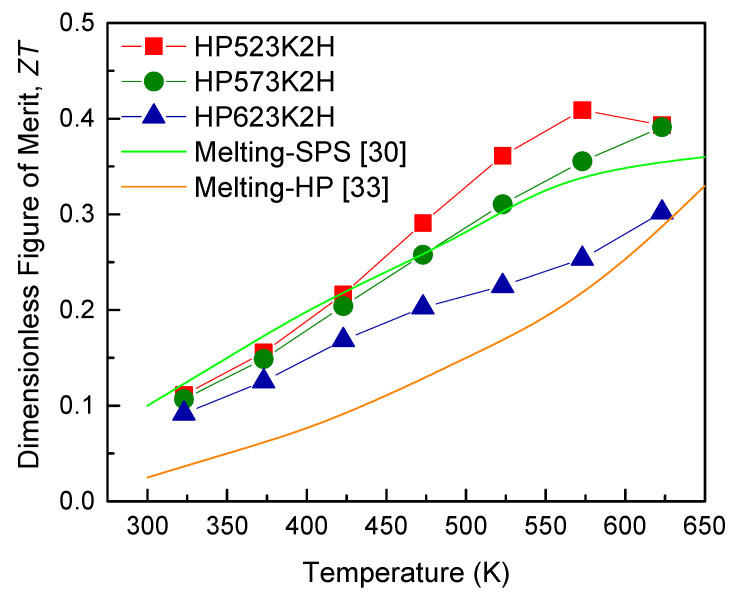
Dimensionless figure of merit for Cu_3_SbSe_4_. SPS: spark plasma sintering.

The *ZT* values of Cu_3_SbSe_4_ are presented in Figure 11. As the temperature increased, the *ZT* value increased, where the maximum *ZT* value was 0.39 at 623 K. Although HP523K2H showed a *ZT* value of 0.41 at 573 K, it is not suitable for thermoelectric applications because of its low relative density. Tyagi et al. [32] and Kumar et al. [30] reported *ZT* values of 0.30 at 550 K and 0.35 at 650 K for Cu_3_SbSe_4_ fabricated via melting and SPS. Tyagi et al. [32] also studied Cu_3_SbSe_3_ (bytizite), which has a composition similar to the permingeatite, and reported a low *ZT* value of 0.04 at 550 K, despite its intrinsically low thermal conductivity (0.26 W m^−1^ K^−1^). Zhang et al. [33] obtained a *ZT* value of 0.33 at 648 K for Cu_3_SbSe_4_ prepared via melting and HP. Bo et al. [34] reported *ZT* values of 0.36 at 625 K and 0.41 at 650 K for Cu_3_SbSe_4_ produced using multi-step processes: melting, annealing, ball-milling, and hot-pressing. Consequently, MA is comparable to the melting process and an effective method for producing Cu_3_SbSe_4_. The optimal HP temperature was determined to be 573 K by considering the sintered density and the thermoelectric properties. It is expected that the thermoelectric performance of Cu_3_SbSe_4_ can be enhanced by reducing the thermal conductivity through the formation of a solid solution and increasing the PF via doping.

## 4. Conclusions

Permingeatite (Cu_3_SbSe_4_) was prepared using MA and HP. The optimal MA condition was determined to be 350 rpm for 12 h from the XRD and TG–DSC analyses. The MA powders were hot-pressed at 523–623 K for 2 h, where sound compacts with high sintered densities were obtained at temperatures above 573 K during HP. The Seebeck and Hall coefficients showed positive signs, indicating *p*-type conduction. The electrical conductivity decreased with increasing HP temperature due to the change in carrier concentration, while the Seebeck coefficient increased. There was minimal charge-carrier contribution to the thermal conductivity. The specimen hot-pressed at 573 K for 2 h exhibited a Seebeck coefficient of 331 μV K^−1^, an electrical conductivity of 4.45 × 10^3^ S m^−1^, a PF of 0.49 mW m^−1^ K^−2^, and a thermal conductivity of 0.76 W m^−1^ K^−1^ at 623 K. As a result, a *ZT* of 0.39 was achieved at 623 K, which was higher than that of Cu_3_SbSe_4_ prepared using the melting process (reported previously). Therefore, the MA–HP methods were confirmed to be practical processes for preparing thermoelectric permingeatite compounds.

## Figures and Tables

**Table 1:** Chemical compositions and physical properties of Cu_3_SbSe_4_.

Specimen	Composition		Relative Density (%)	Lattice Constant (nm)		FWHM_(112)_ (°)	Lorenz Number (10^−8^ V^2^ K^−2^)
	Nominal	Actual		*a*	*c*		
MA350R12H HP523K2H HP573K2H HP623K2H	Cu_3_SbSe_4_	Cu_3.31_Sb_0.97_Se_3.72_ Cu_3.27_Sb_0.93_Se_3.80_ Cu_3.14_Sb_0.97_Se_3.89_ Cu_3.29_Sb_0.93_Se_3.78_	- 89.9 97.8 97.7	0.5651 0.5646 0.5649 0.5650	1.1252 1.1243 1.1243 1.1243	0.355 0.234 0.203 0.171	- 1.62 1.57 1.54

FWHM: full width at half maximum.

## Data Availability

The data presented in this study are available on request from the corresponding author.
